# Molecular Phylogenetic Analysis of Ballistoconidium-Forming Yeasts in Trichosporonales (Tremellomycetes): A Proposal for *Takashimella* gen. nov. and *Cryptotrichosporon tibetense* sp. nov.

**DOI:** 10.1371/journal.pone.0132653

**Published:** 2015-07-22

**Authors:** Long Wang, Qi-Ming Wang

**Affiliations:** State Key Laboratory of Mycology, Institute of Microbiology, Chinese Academy of Sciences, Beijing, China; Leibniz Institute DSMZ-German Collection of Microorganisms and Cell Cultures, GERMANY

## Abstract

*Bullera* species in the Trichosporonales (Tremellomycetes, Agaricomycotina) are phylogenetically distinct from *Bullera alba* (teleomorph: *Bulleromyces albus*), the type species of *Bullera* that belongs to Tremellales. In the present study, the three *Bullera* species, namely *Bullera formosensis*, *Bullera koratensis* and *Bullera lagerstroemiae*, and *Cryptococcus tepidarius* belonging to the Trichosporonales are transferred into a new genus *Takashimella* gen. nov. (MycoBank No. MB 810672) based on sequence analysis of the small subunit (SSU) rRNA gene, the D1/D2 domains of large subunit (LSU) rRNA gene and the ITS+5.8S rRNA gene sequences. In addition, the genus *Cryptotrichosporon* is emended to accommodate a novel ballistoconidium-forming species of the Trichosporonales, which is named as *Cryptotrichosporon tibetense* (type strain CGMCC 2.02614^T^ = CBS 10455^T^). The MycoBank number of this new species is MB 810688.

## Introduction

Ballistoconidium-forming yeasts occur in all currently accepted subphyla of Basidiomycota: Pucciniomycotina, Ustilaginomycotina and Agaricomycotina [1, 2]. The genus *Bullera* was established by Derx [3] to accommodate yeasts producing rotational symmetry of ballistoconidia and whitish to pale colored colonies and proposing *Bullera alba* as the type species for the genus. Stadelmann [4] proposed a new species, namely *Bullera piricola*, to encompass strains producing both symmetrical and asymmetrical ballistoconidia. Since then, the genus *Bullera* has included taxa that form non-pigmented colonies and produce symmetrical or asymmetrical ballistoconidia [5]. Nakase [6] divided the species of *Bullera* into two groups. One group included the species displaying typical morphology of *Bullera*, and the other group (the ‘*Bullera piricola* group’) encompassed the remaining species. The distinctions between the above two groups were based on the morphology of the ballistoconidia and the color of the colonies. However, Nakase [7] suggested that the ‘*Bullera piricola* group’ might represent a genus other than *Bullera*, and this suggestion was later supported by the analysis of the small subunit (SSU) rRNA sequence [8]. Thus, a new genus, *Udeniomyces*, was proposed to accommodate the members of the ‘*Bullera piricola* group’ [9]. The genus *Udeniomyces* is placed in Cystofilobasidiales and not (as other *Bullera*) in Tremellales [10]. Takashima et al. [11] transferred the orange-colored *Bullera* species, *B*. *aurantiaca* and *B*. *crocea*, to *Dioszegia* by emending the genus diagnosis to include ballistoconidium-forming yeasts. Recently, some *Bullera* species phylogenetically closely related to the genus *Dioszegia* were transferred into three new genera: *Derxomyces*, *Hannaella* and *Mingxiaea* [12, 13]. However, although these five genera had been separated from *Bullera*, this genus was still polyphyletic [2, 10, 14–16]. *Bullera* species can still be found in the four orders (Cystofilobasidiales, Filobasidiales, Tremellales and Trichosporonales) of the Tremellomycetes (Agaricomycotina) [2, 16]. In particular, three *Bullera* species, namely *B*. *formosensis*, *B*. *koratensis* and *B*. *lagerstroemiae*, are placed in the Trichosporonales [17, 18]. The three *Bullera* species of Trichosporonales formed a monophyletic group with 100% bootstrap support in a phylogenetic tree based on SSU rRNA sequences [17], and they all produce bilaterally symmetrical ballistoconidia which differ from symmetrical ballistoconidia produced by other species of *Bullera*. Thus, these three *Bullera* species were suggested to be separated from the genus *Bullera* by Fungsin et al. [18]. However, this proposal was not formally completed and was not incorporated in the latest revision of the genus [16].

The order Trichosporonales contains species of the genera *Trichosporon*, *Bullera* and *Cryptococcus* [2, 10, 15]. Apart from the genus *Trichosporon*, member of the genera *Bullera* and *Cryptococcus* should be reclassified in the future as their respective types are placed in Tremellales [2]. For example, the genus *Vanrija* was recently emended and reinstated to include the *Cryptococcus* species in the ‘*humicola*’ clade of the Trichosporonales [19]. The anamorphic genus *Cryptotrichosporon* belonging to Trichosporonales was recently established to accommodate five yeast isolates obtained from cashew tree flowers in Nigeriain [20]. The only known teleomorphic member of the Trichosporonales, a mycoparasite *Tetragoniomyces uliginosus* (former *Tremella uliginosa*) holds a basal position in this order [19, 21].

During our survey of the ballistoconidium-forming yeasts in China, we found two isolates that formed bilaterally symmetrical ballistoconidia and cream-colored colonies and possessed Q-10. The two isolates would be classified in the genus *Bullera* based on the phenotypic characteristics [16]. However, analyses of nucleotide sequence data suggested the relatedness of these cultures with Trichosporonales and not with Tremellales, where the type species of *Bullera* is placed [16]. Thus, classification of these cultures in the genus *Bullera* will further increase its polyphyly. Here, we used three rRNA fragments (the SSU rRNA gene, the D1/D2 domains of LSU rRNA gene and 5.8S rRNA gene) and the internal transcribed spacer (ITS1+ITS2) to analyze the relationships between our two isolates, currently known *Bullera* species and other taxa of Trichosporonales. Our result indicated that the two isolates were phylogenetically more related to *Cryptotrichosporon* than to *Tetragoniomyces* and other genera of Trichosporonales and represent a new member of the genus *Cryptotrichosporon*, which is described here as *Cryptotrichosporon tibetense* sp. nov. In order to reduce the polyphylety of the genus *Bullera* a new genus, *Takashimella* gen. nov., is established to include *B*. *formosensis*, *B*. *koratensis* and *B*. *lagerstroemiae* phylogenetically placed in the Trichosporonales. *Cryptococcus tepidarius* [22], which is phylogenetically related to *B*. *lagerstroemiae*, is also recombined in the genus *Takashimella*.

## Materials and Methods

### Strains and phenotypic characteristics

Two strains, CGMCC 2.02614^T^ (= XZ 20A4 ^T^ = CBS 10455 ^T^) and CGMCC 2.02667 (= XZ 25B1), were isolated from the leaves of *Rhododendron aganniphum* Balf.f. & Kingdon-Ward and *Quercus aquifolioides* Rehd. & Wils., respectively, in Bomi county, Tibet, China, using the method described by Nakase and Takashima [23]. No specific permission was required for collecting specimens in these locations, and the field studies did not involve endangered or protected species. The GPS coordinates of the specific locations used in our study are 29°42′45′′N 95°35′25′′E and 29°40′14′′N 95°29′49′′E. These locations belong to a subtropical climate zone with the annual average temperature of ca. 8.5°C; and the annual precipitation of ca. 900 mm. The examinations of morphological, physiological and biochemical characteristics of the strains followed the methods [24]. Assimilation of carbon and nitrogen compounds were investigated on liquid media [24]. The ubiquinones tests were performed according to Yamada and Kondo [25]. The type culture of *Cryptotrichosporon tibetense* sp. nov. was deposited in the China General Microbiological Culture Collection (CGMCC) of the Institute of Microbiology at the Chinese Academy of Sciences, Beijing, China, as CGMCC 2.02614^T^. A ex-type strain of *Cryptotrichosporon tibetense* was deposited in the Centraalbureau for Schimmelcultures (CBS) of the Royal Netherland Society of Sciences in Utrecht as CBS 10455^T^. All cultures are also maintained at the corresponding author's laboratory and will be supplied upon request for educational or scientific purposes.

### Molecular phylogenetic analysis

The PCR amplification and sequencing of the ITS1-5.8S-ITS2 and D1/D2 domains of the LSU rRNA gene were performed using previously described methods [26]. The SSU rRNA gene sequences were obtained using the method employed by Wang et al. [27]. Sequences were aligned with the MAFFT program V7.130b using the L-INS-I algorithm [28]. The CADM test (Congruence Among Distance Matrices test) [29] included in MLSTest [30] was used to analyze the congruence between these three gene regions with 10000 permutations, all other parameters were settled as default. The combined three-gene dataset was first analyzed with jModeltest [31] using the Akaike information criterion to find the most appropriate model for DNA substitution. A general time-reversible model of DNA substitution that assumes a percentage of invariable sites and *Γ*-distributed substitution rates at the remaining sites (GTR + I + G) was selected for further analyses (AIC = 32362.5580). A phylogenetic tree was constructed by the maximum likelihood (ML) in RAxML-HPC2 7.2.8 [32] with a rapid bootstrap analysis using a random starting tree and 1000 bootstrap replicates searching for the best maximum-likelihood tree, and GTRGAMMAI was used as the model of evolution. Maximum parsimony (MP) analysis was conducted in PAUP* 4.0b10 [33] with a heuristic search with 1000 random additions and TBR. Bootstrap analyses were performed from 1000 replicates using 10 random additions and TBR for each replicate. The gaps in the alignment were treated as missing data. MulTrees and Steepest descent options were not in effect. Bayesian inference (BI) was conducted in MrBayes 3.2 [34] with GTR + I + G model and parameters set to 5000000 generations with two independent runs and four chains starting with random trees. Trees were sampled every 1000 generations leading to an overall sampling of 5000 trees. The analysis was stopped when the standard deviation of split frequencies between the trees generated in the independent runs was below 0.01. 25% of these trees were discarded, the remaining were used to compute a 50% majority rule consensus tree to obtain estimates for posterior probabilities.

## Results and Discussion

**Fig 1:**
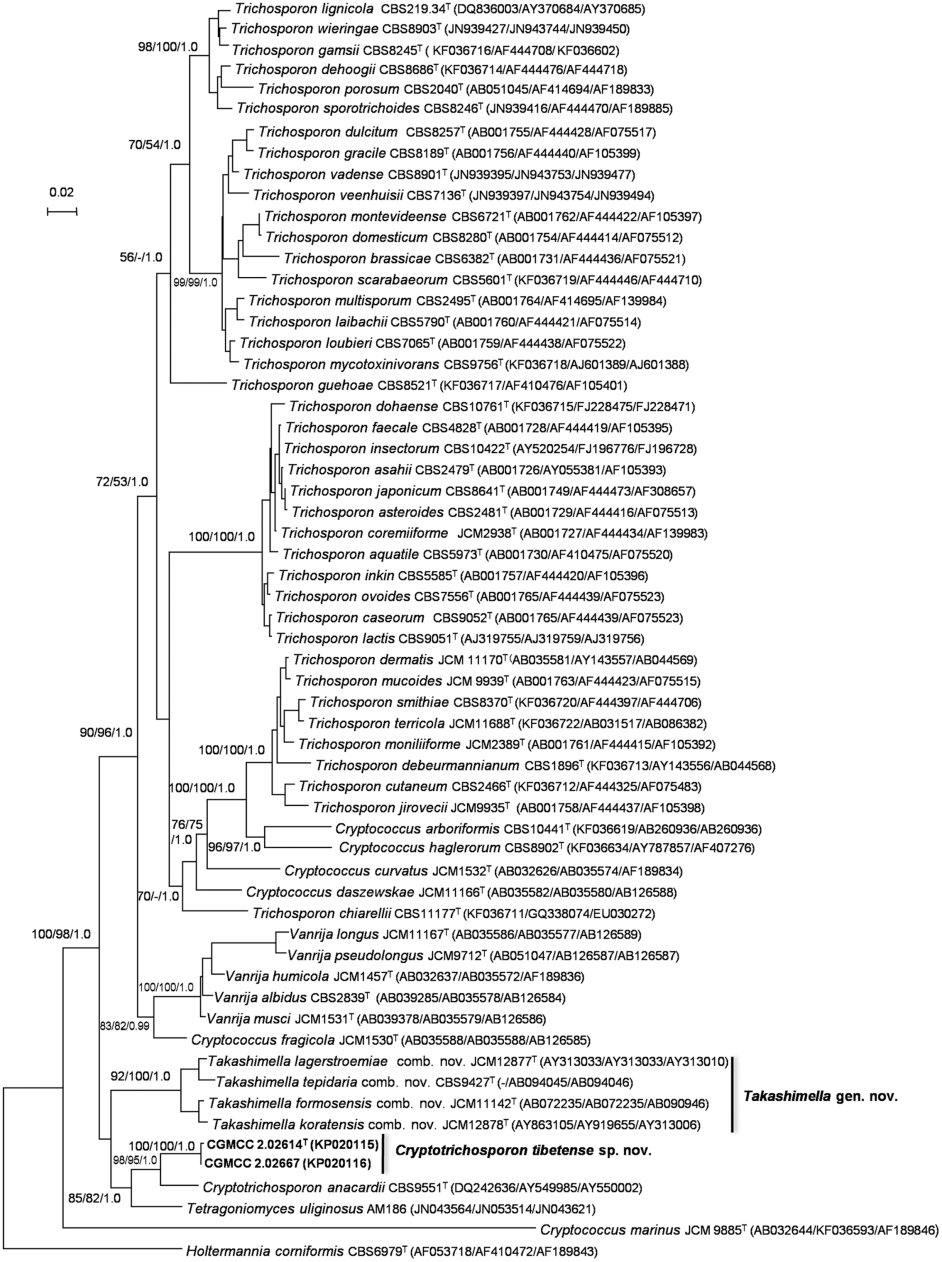
The combined three gene regions ML tree. The best tree found in maximum-likelihood analysis of the combined sequences of the SSU rRNA, the LSU rRNA D1/D2 domains and the ITS region (including the 5.8S rRNA) depicting the relationships between the *Bullera* species and other clades within Trichosporonales (Tremellomycetes). Bootstrap percentages over 50% from the 1000 bootstrap replicates and Bayesian posterior probabilities above 0.9 are shown. Bar = 0.02 substitutions per nucleotide position.

The MAFFT algorithm [28] was used to align the sequences of the SSU rRNA gene, D1/D2 domains of LSU rRNA gene and ITS+5.8S rRNA gene, and resulted in alignments of 1654 nucleotides, 633 nucleotides and 522 nucleotides, respectively. The congruence between these three gene regions was analyzed using CADM [29, 30] and resulted an incongruence level of *p* = 0.0001. A Kendall’s value of W = 0.7106 indicated that these three gene regions could be considered as congruent [29, 35]. Thus, these three gene regions were combined as one dataset for further analyses. The final combined three-gene super-matrix consisted of 2809 nucleotides (S1 Dataset). Three trees constructed by ML, MP and BI methods had the visually similar topology. Thus, ML tree was used in this study (Fig 1). The combined analyses of the three gene regions showed that our two isolates (CGMCC 2.02614^T^ and CGMCC 2.02667) producing bilaterally symmetrical ballistoconidia clustered with *Cryptotrichosporon anacardii* with 1.0 PP (posterior probability from Bayesian inference) and 95–98% BP (bootstrap percentages from ML and MP analyses) support (Fig 1). *B*. *formosensis*, *B*. *koratensis*, *B*. *lagerstroemiae* and *Cryptococcus tepidarius* formed a well-supported clade that also had 1.0 PP and 92–100% BP support (Fig 1). *Tetragoniomyces uliginosus* clustered with the genus *Cryptotrichosporon* with 82–85% BP and 1.0 PP support in our analysis (Fig 1). These three clades formed basal lineages in the Trichosporonales (Fig 1).

*B*. *formosensis* was described by Nakase et al. [17] based on one strain. It contained intracellular xylose, possessed Q-10 as a major component of its ubiquinones, and produced ballistoconidia and budding cells. However, this species was distant from other members of the genus *Bullera* in a phylogenetic tree based on the nucleotide sequences of the SSU rRNA gene, and was instead located in the Trichosporonales [17]. Fungsin et al. [18] identified two new ballistoconidium-forming yeast species in the Trichosporonales: *B*. *lagerstroemiae* and *B*. *koratensis*. These two *Bullera* species formed a well separated cluster with *B*. *formosensis* that was distant from other clusters in the Trichosporonales [18]. Boekhout et al. [16] suggested that these three species should be reclassified as one new genus from the type species of *Bullera*, viz., *B*. *alba* (teleomorph *Bulleromyces albus*), which belongs to Tremellales. *Cryptococcus tepidarius* was described by Takashima et al. [22] based on two thermotolerant and acid-tolerant strains isolated from a hot-spring area in Japan. This species was phylogenetically closely related to *B*. *lagerstroemiae* and formed the distinct *B*. *formosensis* clade with *B*. *formosensis*, *B*. *koratensis* and *B*. *lagerstroemiae* in the Trichosporonales. This clade appeared to be closely related to *Cryptotrichosporon*, as demonstrated in the analysis made by Okoli et al. [20] even though bootstrap support value in the LSU rRNA D1/D2 domains tree was low (56%) for this group. *Cryptococcus thermophilus* was described in the Trichosporonales based on the D1/D2 domains of LSU rRNA gene sequence analysis [36]. Only the LSU rRNA D1/D2 domains sequence of *C*. *thermophilus* is available in public databases. Thus, this species was not included in our combined three-gene analysis. But a LSU rRNA D1/D2 domains ML tree (S1 Fig) was constructed to show the phylogenetic relationships between this species and the other species including our two isolates in the Trichosporonales. In this analysis, *C*. *thermophilus* was closely related to *Vanrija* spp. that is in agreement with Vogelmann et al. [36].

The genus *Bullera* is polyphyletic [1, 2, 10, 15, 16]. The majority of the species of *Bullera* including the generic type *B*. *alba* belong to the Tremellales, whereas species of the *B*. *formosensis* clade occur in the Trichosporonales. In order to reduce the polyphylety of the genus *Bullera* we follow the principle of an evolution-based classification using monophyly as the leading concept [20] and propose the designation of a new genus, namely *Takashimella* gen. nov., to accommodate the species in the *B*. *formosensis* clade. Our two isolates (CGMCC 2.02614^T^ and CGMCC 2.02667) form bilaterally symmetrical ballistoconidia as the *Bullera* species of the Trichosporonales do. They are more closely phylogenetically related to *Cr*. *anacardii* than to the *B*. *formosensis* clade and *Tetragoniomyces uliginosus* according to our sequence analysis (Fig 1). The two isolates differ from *Cr*. *anacardii* by 20 (4%) and 38 (7%) nucleotides mismatch in the D1/D2 domains of LSU rRNA gene and ITS region, respectively. *T*. *uliginosus* was phylogenetically related to the genus *Cryptotrichosporon* with moderate support values (82–85% BP), however, the genus *Cryptotrichosporon* and our two isolates formed a strong support (95–98% BP) clade (Fig 1). *T*. *uliginosus* formed a single species lineage distant from the genus *Cryptotrichosporon* in the Bayesian tree constructed from the combined SSU, 5.8S and LSU rRNA genes dataset [21]. This species showed some relatedness to the genus *Cryptotrichosporon* in analysis of the D1/D2 domains of LSU gene, but lacked statistical support [20]. *T*. *uliginosus* formed a separated branch in the LSU rRNA D1/D2 domains tree in this study (S1 Fig) in agreement with Millanes et al. [21]. In our opinion, it is better to classify our two isolates in the genus *Cryptotrichosporon* than to combine the two isolates and the members of the two genera *Cryptotrichosporon* and *Tetragoniomyces* into one genus. Here, we emend the genus *Cryptotrichosporon* to accommodate the two ballistoconidium-forming isolates and describe them here as a new species *Cryptotrichosporon tibetense* sp. nov.

### Nomenclature

The electronic version of this article can be obtained in Portable Document Format (PDF) using the ISSN or ISBN. This article represents a published work according to the International Code of Nomenclature for algae, fungi, and plants. The new names contained in the electronic publication of a PLOS ONE article are effectively published under this nomenclature code from the electronic edition alone; there is therefore no longer any need to provide printed copies.

In addition, the new names contained in this work have been submitted to MycoBank, from where they will be made available to the Global Names Index. The unique MycoBank number can be resolved and the associated information viewed through any standard web browser by appending the MycoBank number contained in this publication to the prefix http://www.mycobank.org/MB/. The online version of this work is archived and available from the following digital repositories: PubMed Central and LOCKSS.

### Description of *Takashimella* Q. M. Wang gen. nov. Fig 1

[urn:lsid:indexfungorum.org:names: 810672], MycoBank MB 810672

Etymology: This genus is named in honor of Masako Takashima, Biological Resource Center (NBRC), Department of Biotechnology, National Institute of Technology and Evaluation, Japan, for her numerous contributions to the taxonomy of yeasts.

This genus belongs to Trichosporonales, Tremellomycetes, Agaricomycotina, which is phylogenetically circumscribed from the analysis of the SSU rRNA gene, the D1/D2 domains of LSU rRNA gene and the ITS+5.8S rRNA gene sequences (Fig 1). The colonies are butyrous, dull, smooth or wrinkled, cream or pale yellowish-brown in color, and they have an entire or undulate margin. Hyphae and pseudohyphae may be formed. The yeast cells are ovoid, subglobose, cylindrical or ellipsoidal. Budding is polar. Ballistoconidia are ellipsoidal and kidney- to comma-shaped, if formed. Diazonium blue B (DBB) reaction and urea hydrolysis are positive. Xylose is found in the cell-wall hydrolysates. The major ubiquinone is CoQ 10. Production of starch-like compounds is variable. Sexual reproduction has not been observed. All currently known species of this genus can assimilate sucrose and raffinose, whereas the members of the sister genus *Cryptotrichosporon* can not use either of the two carbon sources. The genera *Takashimella* and *Cryptotrichosporon* can be well distinguished from each other by the above physiological tests as well as by the phylogenetic analysis of three ribosomal gene regions (Fig 1).

Type species: *Takashimella formosensis* (Nakase, Tsuzuki & Takashima) Q.M.Wang comb. nov.

[urn:lsid:indexfungorum.org:names: 810673], MycoBank MB 810673

Basionym: *Bullera formosensis* Nakase, Tsuzuki & Takashima, J Gen Appl Microbiol, 48: 345, 2002[MB#484489].

### New combinations of *Takashimella*

#### Takashimella koratensis

(Fungsin, Takashima, Sugita & Nakase) Q. M. Wang **comb. nov.**

[urn:lsid:indexfungorum.org:names: 810674], MycoBank MB 810674

Basionym: *Bullera koratensis* Fungsin, Takashima, Sugita & Nakase, J Gen Appl Microbiol, 52: 73, 2006 [MB#510194].

#### Takashimella lagerstroemiae

(Fungsin, Takashima, Sugita & Nakase) Q. M. Wang **comb. nov.**

[urn:lsid:indexfungorum.org:names: 810686], MycoBank MB 810686

Basionym: *Bullera lagerstroemiae* Fungsin, Takashima, Sugita & Nakase, J Gen Appl Microbiol, 52: 73, 2006[MB#510193].

#### Takashimella tepidaria

(Takashima, Sugita, Toriumi & Nakase) Q. M. Wang **comb. nov.**

[urn:lsid:indexfungorum.org:names: 810687], MycoBank MB 810687

Basionym: *Cryptococcus tepidarius* Takashima, Sugita, Toriumi & Nakase, Int J Syst Evol Microbiol, 59: 181, 2009 [MB#514899].

### Emendation of *Cryptotrichosporon* (Okoli & Boekhout) FEMS Yeast Res,7: 348 (2007) emend. Q. M. Wang

This genus is circumscribed based on the analysis of the SSU rRNA gene, the D1/D2 domains of LSU rRNA gene and the ITS+5.8S rRNA gene sequences (Fig 1) and is emended to include ballistoconidium-forming and non ballistoconidium-forming yeasts in the *Cryptotrichosporon* clade of Trichosporonales (Fig 1).

The colonies are cream to pale and yellowish-brown, smooth, mucoid, shiny. They have full margins. The yeast cells are ovoid to ellipsoid; pseudohyphae are not formed. Budding is polar. Ballistoconidia may be present and are falcate or amygdaliform. Diazonium blue B (DBB) reaction and urea hydrolysis are positive. The major ubiquinone is CoQ 10. Production of starch-like compounds is variable, which is different from the original diagnosis by Okoli et al. [20]. Sexual reproduction has not been observed. Phylogenetic analysis (Fig 1) indicated the relatedness of the genus *Cryptotrichosporon* with the teleomorph species *Tetragoniomyces uliginosus*, suggesting possible mycoparasitic lifestyle in this group.

### Description of *Cryptotrichosporon tibetense* Q. M. Wang sp. nov. Figs 1, 2

[urn:lsid:indexfungorum.org:names: 810688], MycoBank MB 810688

Etymology: The specific epithet *tibetense* (tibet. en'se N.L. neut. adj. tibet pertaining to Tibet; referring to the geographical origin of the type strain of this species).

**Fig 2:**
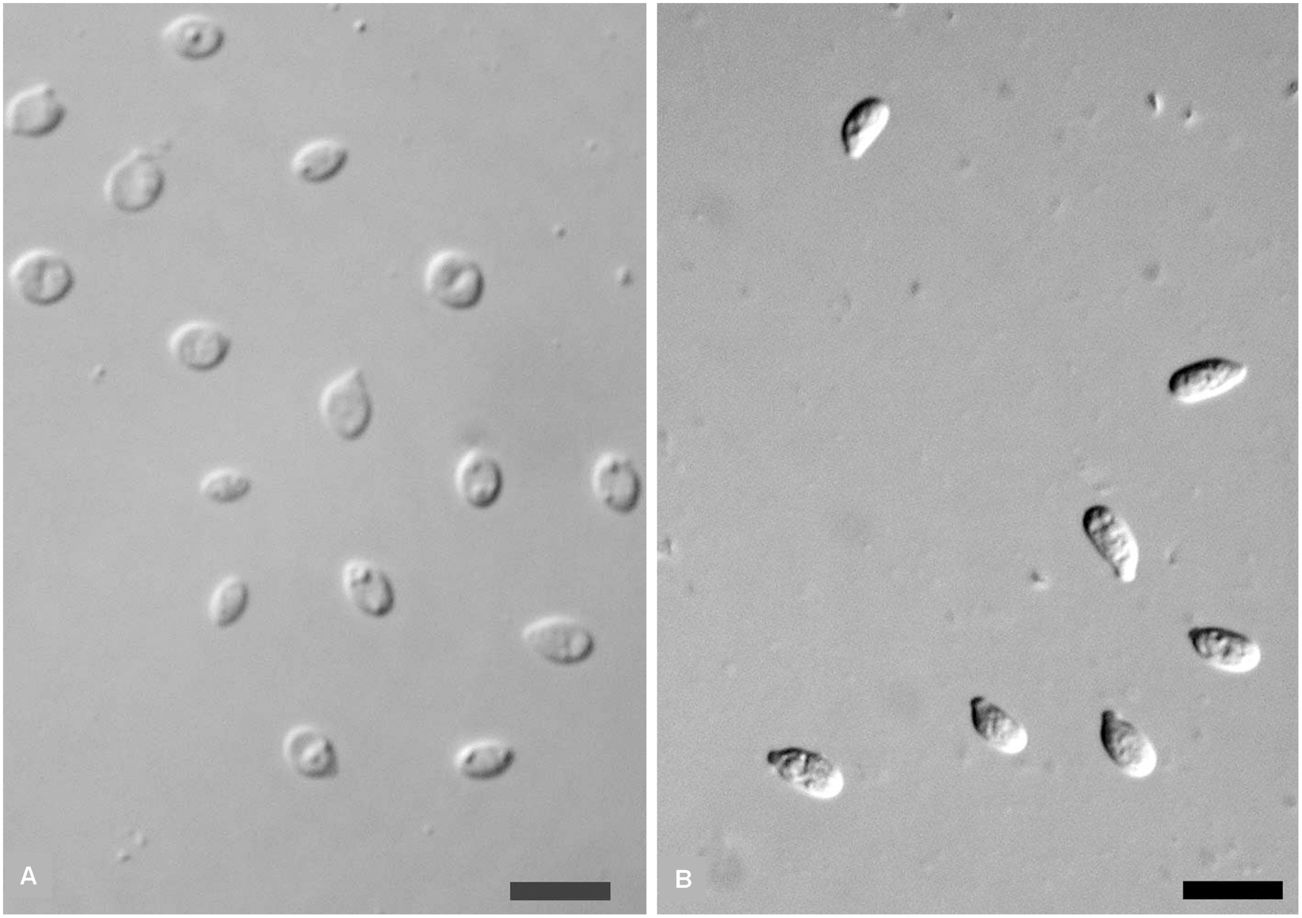
Cells and ballistoconidia. Morphology of *Cryptotrichosporon tibetense* CGMCC 2.02614^T^. A, vegetative cells in YM broth for 7 days at 17°C; B, ballistoconidia produced on corn meal agar after 7 days at 17°C. Bars = 10 μm.

After culturing for 7 days at 17°C in YM broth, the cells are ovoid or ellipsoidal and are 2.5–5.2 × 4.8–8.0 μm in dimension (Fig 2). The budding is polar, and a sediment is formed. After 1 month at 17°C a ring and sediment are present. After 1 month at 17°C on YM agar, the streak culture is cream-colored, butyrous, and smooth. The margin is complete. In the Dalmau plate culture on corn meal agar, pseudohyphyae are not formed. Ballistoconidia are turbinate, and their dimensions are 3.0–4.5 × 6.0–9.0 μm (Fig 2). Fermentation is negative. Glucose, maltose, cellobiose, trehalose, D-xylose, L-arabinose and D-mannitol are assimilated. Melezitose and D-glucitol are assimilated, but delayed and weakly. Sucrose, galactose, L-sorbose, lactose, melibiose, raffinose, inulin, L-rhamnose, glycerol, D-arabinose, D-ribose, methyl α-D-glucoside, methanol, ethanol, erythritol, galactitol, DL-lactic acid, salicin, critic acid, succinic acid, inositol and hexadecane are not assimilated. Assimilation of soluble starch, D-glucosamine and ribitol are variable. Ammonium sulfate, potassium nitrate, sodium nitrite (variable), L-lysine, ethylamine hydrochloride and cadaverine dihydrochloride are assimilated. The maximum growth temperature is 26°C. Growth in vitamin-free medium is positive. Starch-like substances are not produced. Growth on 50% (w/w) glucose-yeast extract agar is negative. Urease activity is positive. The Diazonium Blue B reaction is positive, and the major ubiquinone is Q-10.

The type culture CGMCC 2.02614^T^ is from China: Tibet, Bomi county, from 29°42′45′′N 95°35′25′′E, 2822 m. Specimens were collected from leaves of *Rhododendron aganniphum* in July of 2004: coll. Feng-Yan Bai, leaf sample no. XZ20. The additional culture CGMCC 2.02667 is from China: Tibet, Bomi county, 29°40′14′′N 95°29′49′′E, 2150 m, from leaves of *Quercus aquifolioides*, July, 2004, coll. Feng-Yan Bai, leaf sample no. XZ25.

## Supporting Information

S1 Dataset The alignment of three rDNA gene regions. The alignment of the combined SSU, D1/D2 domains of LSU and ITS+5.8S rRNA sequences.
(TXT)

S1 Fig The D1/D2 ML tree. The maximum-likelihood analysis of the D1/D2 domains of LSU rRNA, depicting the relationships of these taxa in the Trichosporonales. Bootstrap percentages over 50% from the 1000 bootstrap replicates are shown. Bar = 0.05 substitutions per nucleotide position.
(TIF)
