# Supplementary figures and images for: Molecular Phylogenetic Analysis of Ballistoconidium-Forming Yeasts in Trichosporonales (Tremellomycetes): A Proposal for Takashimella gen. nov. and Cryptotrichosporon tibetense sp. nov
Source: PLoS One. 2015 Jul 22;10(7):e0132653. doi: 10.1371/journal.pone.0132653 (PMC4511645; doi:10.1371/journal.pone.0132653)

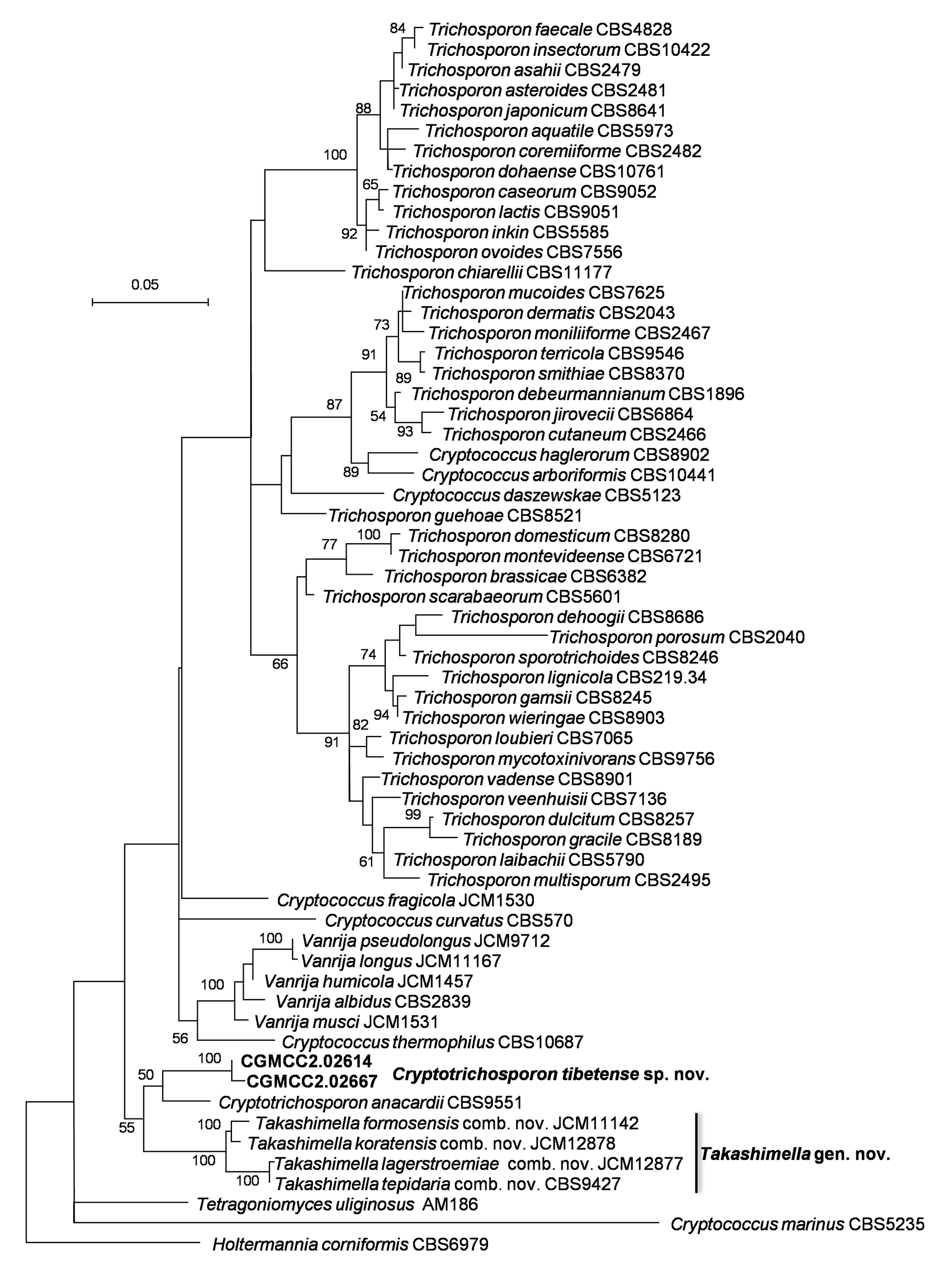

Supplement: S1 Fig — The maximum-likelihood analysis of the D1/D2 domains of LSU rRNA, depicting the relationships of these taxa in the Trichosporonales. Bootstrap percentages over 50% from the 1000 bootstrap replicates are shown. Bar = 0.05 substitutions per nucleotide position. (TIF) [file pone.0132653.s002.tif]
